# Seroprevalence of hepatitis E virus among blood donors on Corsica, France, 2017

**DOI:** 10.2807/1560-7917.ES.2020.25.5.1900336

**Published:** 2020-02-06

**Authors:** Lisandru Capai, Nathanaël Hozé, Jacques Chiaroni, Sylvie Gross, Rachid Djoudi, Rémi Charrel, Jacques Izopet, Frédéric Bosseur, Stéphane Priet, Simon Cauchemez, Xavier de Lamballerie, Alessandra Falchi, Pierre Gallian

**Affiliations:** 1 EA 7310, Laboratoire de Virologie, Université de Corse, Corte, France; 2 Mathematical Modelling of Infectious Diseases Unit, Institut Pasteur, UMR2000, CNRS, Paris, France; 3 Etablissement Français du Sang Provence alpes Côte d’Azur et Corse, Marseille, France; 4 Etablissement Français du Sang, 93210, La Plaine-Saint-Denis, France; 5 Unité des Virus Émergents (UVE): Aix Marseille Univ, IRD 190, INSERM 1207, IHU Méditerranée Infection, Marseille, France; 6 Laboratoire de Virologie, Institut Fédératif de Biologie, Centre Hospitalier et Universitaire, Toulouse, France; 7 Institut National de la Santé et de la Recherche Médicale Unité 1043, Université Toulouse III, Toulouse, France; 8 Sciences Pour l’Environnement – UMR CNRS 6134 Université de Corse, Corte, France

**Keywords:** Hepatitis E virus, Epidemiology, IgG titres, Seroprevalence, Blood donors, Corsica

## Abstract

**Background:**

Hepatitis E virus (HEV) is an emerging zoonotic pathogen and an important cause of acute viral hepatitis in European countries. Corsica Island has been previously identified as a hyperendemic area for HEV.

**Aim:**

Our aim was to characterise the prevalence and titres of IgG antibodies to HEV among blood donors on Corsica and establish a model of the annual force of infection.

**Methods:**

Between September 2017 and January 2018, 2,705 blood donations were tested for anti-HEV IgG using the Wantai HEV IgG enzyme immunoassay.

**Results:**

The overall seroprevalence was 56.1%. In multivariate analysis, seroprevalence was higher in men than in women (60.0% vs 52.2%; p < 0.01), increased with age and was significantly higher among donors born on Corsica (60.6% vs 53.2%; p < 0.01). No significant difference was observed between the five districts of the island. IgG anti-HEV titres were mostly low (70% of positive donors had titres < 3 IU/mL). In Corsican natives, increasing seroprevalence by age could be explained by models capturing a loss of immunity (annual probability of infection: 4.5%; duration of immunity: 55 years) or by age-specific probabilities of infection (3.8% for children, 1.3% for adults).

**Conclusion:**

We confirmed the high HEV seroprevalence on Corsica and identified three aspects that should be further explored: (i) the epidemiology in those younger than 18 years, (ii) common sources of contamination, in particular drinking water, that may explain the wide exposure of the population, and (iii) the actual protection afforded by the low IgG titres observed and the potential susceptibility to secondary HEV infection.

## Introduction

Hepatitis E virus (HEV) is a virus with a single-stranded positive-sense RNA of ca 7.5 kb and belongs to the family *Hepeviridae* (genus *Orthohepevirus*) [1]. HEV was originally described as a non-enveloped virus [2,3] but a quasi-enveloped form has been observed in blood [4,5]. HEV strains infecting humans are currently classified in five genotypes (HEV-1 to HEV-4 and HEV-7) [6] but belong to a single serotype.

HEV-1 and 2 infections have been reported in humans, with person-to-person transmission occurring via the faecal-oral route. These strains are responsible for both epidemics and sporadic cases in low and middle income countries. HEV-3 and 4 are zoonotic pathogens infecting humans and domestic (e.g. pigs) and wild animal species (e.g. boars, deer) which constitute the animal reservoir [7]. The main route of infection for genotypes 3 and 4 is the consumption of contaminated food or water or the contact with infected animals [1,8]. Genotypes 3 and 4 are responsible for sporadic autochthonous cases in high-income countries.

HEV infection can lead to acute hepatitis but can also, in a considerable proportion of cases, be asymptomatic (or at least present in a way that does not call medical attention) [9-11]. Severe presentations (fulminant hepatitis) have been reported when infection occurs during pregnancy [12,13]. In low and middle income countries, chronic forms of infection have been reported in immunocompromised patients [14-17] or those with pre-existing liver diseases [18] infected by HEV genotype 3 [19,20] or genotype 4 [17,21]. 

Interpretation and comparison of the seroprevalence studies remain difficult because sensitivity and specificity rates of the different anti-HEV IgG assays vary widely [22,23]. The Wantai assay is frequently used in European countries [24] and has specificity and sensitivity values for HEV IgG in the order of magnitude of, respectively, 93.2% and 97.8% [25-27]. This test is based on a recombinant antigen corresponding to ORF2, which encodes the structural protein of the icosahedral capsid [28]. Epidemiological studies in Europe, conducted with the Wantai serological test, showed great geographical heterogeneity of anti-HEV carriers with seroprevalences ranging from 5–6% in Scotland [29,30] to 52% in south-western France [31]. Important variability was observed even within individual countries [32-34]. A nationwide seroprevalence study conducted in France reported a 22% anti-HEV IgG seroprevalence in the French blood donor population tested in 2011 and 2012 [32]. In this study, seropositivity was associated with male sex, increasing age, and consumption of oysters, pork meat and raw pork liver sausages. Drinking bottled water was associated with lower anti-HEV IgG seropositvity rate. A national report on the surveillance of HEV in France has shown an increase in the number of autochthonous cases detected between 2002 and 2016 (from nine to 2,292) [35]. However, this increase could be due to awareness among physicians and in the general population, resulting in increased testing [35]. 

With a population of 338,000 inhabitants in 2018 [36], Corsica is a large French island (8,680 km^2^) located in the Mediterranean Sea in the south-east of mainland France. The aim of the study was to investigate HEV seroprevalence in volunteer blood donors from Corsica, an area considered as a zone of high prevalence [32] and to assess the level of immunity in the population together with a fine-scale analysis of prevalence in Corsican districts.

## Methods

### Studied population

Blood donors accepted for donation according to the national requirements and living on Corsica were included in the study from September 2017 to January 2018. Only five of 2,710 eligible donors (0.18%) did not consent that their samples might be used for epidemiological studies and were excluded; accordingly the population studied provided a good picture of the population of blood donors in Corsica. French (including Corsican) blood donors are healthy volunteers 18 to 70 years-old. This population, when compared with the general population, presents well-known representativeness biases: children and young adults (< 18-years-old) are not represented, the elderly population (> 65-years-old) is underestimated and blood donors are healthy. Such biases have never been associated in the age class of recruitment with a significant distortion of sero-epidemiological results of infectious diseases in comparison with the general population, as long as the analysis does not imply agents of chronic diseases disqualifying for blood donation. Information about the distribution of the studied population and the general population of Corsica by age group and sex is available in Supplementary Figure S1.

Only data recorded in the database of the French Public Transfusion service (Établissement Français du Sang: EFS) (age, sex, place of residence and birth) were used in the study after anonymisation. There was no specific epidemiological questionnaire for this study, we used the regular blood donation questionnaire that is identical in all French territories (this questionnaire assesses donor health, travel history as well as risk factors for the donor and the recipient such as blood borne infections and medications). Blood donors born in or outside Corsica were assigned to the ‘native’ or ‘non-native’ group, respectively.

### Laboratory methods

#### Anti-HEV IgG detection

Plasma samples were tested for the presence of anti-HEV IgG (EFS Provence Alpes Cotes d’Azur and Corse, Marseille) using a single lot reagent of the Wantai HEV IgG enzyme immunoassay kit (Wantai Biologic Pharmacy Enterprise, Beijing, PRC). The assay is based on a recombinant antigen encoded by a structural region of ORF-2 derived from a Chinese isolate of HEV-1 [28,37] and its analytical and clinical performances have been evaluated recently [38]. For each sample, we calculated the ratio sample OD/cut-off OD. A result was considered positive if the sample ratio was ≥ 1. The limit of detection of the Wantai IgG anti-HEV assay is 0.25 IU/mL and was determined using World Health Organization (WHO) standards [39].

#### Anti-HEV IgG quantification

Anti-HEV IgG titres were estimated as previously described [40] using dilution series (ranging from 0.25 to 5.0 IU/mL) from the WHO Anti-HEV IgG reference material, standard 95/584 (supplied by the National Institute for Biological Standards and Control, South Mimms, United Kingdom (UK)). For samples with titres > 5.0 IU/mL, final titre was determined after testing dilutions of these samples until the signal was in the linearity part of the titration curve (0.25–5.0 IU/mL)

#### Blood grouping

ABO, Rhesus and Kell blood groups were determined using a fully automated microplate haemagglutination procedure [41] according to routine EFS procedures.

### Statistical analysis

Association of the presence of anti-HEV IgG with biological factors (blood groups) and epidemiological data (sex, age, place of residence and place of birth) was analysed and tested using Fisher’s exact test. All variables with a p value < 0.2 were included in the multivariate analyses using an unconditional logistic regression model. Statistical significance was set at a p value < 0.05. Multivariate analysis was performed using an unconditional logistic regression model. Results were considered statistically significant when the p value was lower than 0.05. All statistical analyses were performed using R software version 3.6.1 (R Foundation, Vienna, Austria).

#### Models of the annual force of infection

The analysis of seroprevalence stratified by age can provide insight about the history of circulation of HEV on Corsica, the risk factors associated with exposure as well as patterns of long-term antibody decay following HEV infection. Indeed, serocatalytic models are now commonly used to reconstruct from such data trends in the force of infection *λ_a_*, defined as the rate of infection among susceptible individuals of age *a* [42]. For example, if the force of infection is constant with age (*λ_a_* = *λ*) and if there is no decay in immunity, these models predict that the annual probability of infection for a susceptible individual is 1 − *exp*(−*λ*) and the seroprevalence in individuals of age *a* is *P_a_* = 1 − *exp*(−*λ × a*). By fitting model predictions to data, it is possible to estimate the force of infection.

Here, we considered three competing serocatalytic models to explain the observed age-stratified seroprevalence. Firstly, we considered the simple model described above where the force of infection is age-invariant (*λ_a_* = *λ*) and there is no decay in immunity. Secondly, we assumed that the force of infection is different for children (*λ_c_*; < 18-years-old) and for adults (*λ_A_*), still assuming no decay in immunity. In this second model, the seroprevalence in individuals of age *a* is:

Finally, we assumed that the force of infection is age-invariant but immunity decays at a rate *ρ*:



In the third model, men and women can have a different risk of infection but are assumed to have the same duration of immunity.

The contribution to the likelihood of an individual of age *a* with seropositivity status *s* (*s* = 1 if seropositive; otherwise *s* = 0) is (*P_a_*)^s^ (1 − *P_a_*)^1 −^ *^s^*. Parameters were estimated in a Bayesian framework using a Markov Chain Monte Carlo (MCMC) method implemented in R using the Rstan package [43]. Four independent chains of 5,000 iterations each were simulated, with the first 2,500 iterations corresponding to the burn-in. We chose flat priors for the force of infection *λ* and the seroreversion rate *ρ*.

Means and 95% credible intervals (CrI) of the posterior distributions were calculated. We report the annual probability of infection of a susceptible individual using the formula *p* = 1 − *e* ^−λ^. The seroreversion rate was converted into a duration of immunity using the expression: *d* = 1/*ρ_._*

Goodness of fit of the different models was assess using the deviance information criterion (DIC), a measure of the deviance in the likelihood commonly used in parameter estimations in a Bayesian setting. The smaller the DIC of a model, the better the model fit [44]. A DIC difference > 4 corresponds to a substantial improvement of fit.

### Ethical statement

The study received approval from the medical and scientific direction of the French Public Transfusion service EFS and from the ad hoc ethics committee (Comité de Protection des Personnes #2016-A01000–51). All French blood donors are voluntary and non-remunerated. Blood donors were informed that samples might be used for epidemiological studies. Donors who did not consent were not included.

## Results

### Anti-HEV IgG seroprevalence analyses

#### Study population

A total of 2,705 blood donors (18–70-years-old) living on Corsica for at least 6 months participated in this study, including 1,347 women (49.80%) with a mean age of 42 years and 1,358 men (50.20%) with a mean age of 43 years (Table 1).

**Table 1:** Univariate analysis of biological and epidemiological factors associated with IgG antibodies to hepatitis E virus, Corsica, 2017 (n = 2,705)

Parameters	Variables	n	Anti-HEV IgG-positive		Variable	p value	OR (95% CI)
			**n**	**%**			
**Sex**	Women	1,347	703	52.19	Men vs women	0.000024	1.37 (1.18–1.60)
	Men	1,358	815	60.01			
**Age group (years)**	18–27	605	278	45.95	One age group vs all other age groups	< 0.00001	0.59 (0.49–0.70)
	28–37	492	244	49.59		0.0008	0.72 (0.59–0.88)
	38–47	575	323	56.17		NS	NS
	48–57	566	377	66.61		< 0.0001	1.74 (1.44–2.12)
	58–70	467	296	63.38		0.0003	1.44 (1.17, 1.77)
**Place of residence (district)**	Ajaccio	1,047	611	58.36	One district vs all other districts	0.023	1.18 (1.01–1.37)
	Bastia	560	304	54.29		NS	NS
	Calvi	258	140	54.26		NS	NS
	Corte	419	223	53.22		NS	NS
	Sartène	421	240	57.01		NS	NS
**Native of Corsica**	Yes	1,068	647	60.58	Native vs non-native	< 0.0001	1.35 (1.16–1.58)
	No	1,637	871	53.21			
**ABO blood group**	A	1,019	569	55.84	One BG vs all other BG	NS	NS
	B	257	136	52.92		NS	NS
	AB	105	65	61.90		NS	NS
	O	1,324	748	56.50		NS	NS
**Rhesus D**	Positive	2,250	1,261	56.04	Rh+ vs Rh−	NS	NS
	Negative	455	257	56.48			
**Kell**	Positive	238	135	56.72	Kell+ vs Kell−	NS	NS
	Negative	2,467	1,383	56.06			

BG: blood group; CI: confidence interval; HEV: hepatitis E virus; NS: non-significant value; Rh: Rhesus factor.

#### Seroprevalence

The overall seroprevalence for anti-HEV IgG antibodies in this population was 56.1% (95% confidence interval (CI): 54.2–58.0). The adjusted estimate of seroprevalence in the general population of Corsica in the same age group was 55.8% (95% CI: 53.9–57.7; see Supplementary Table S1).

#### Sex

In both univariate and multivariate analysis (Table 1 and 2), the seroprevalence was significantly higher in male (60.0%) than in female (52.2%) donors (p value < 0.001). Significance was lost when the analysis was restricted to native individuals.

**Table 2:** Multivariate analysis of biological and epidemiological factors associated with IgG antibodies to hepatitis E virus, Corsica, 2017 (n = 2,705)

Parameters	Variable	p value	AOR (95% CI)
**Sex**	Men vs women	0.002	1.28 (1.12- 1.46)
**Age group (years)**	18–27	< 0.00001	0.60 (0.49–0.73)
	28–37	0.018	0.74 (0.60–0.91)
	48–57	0.00021	1.68 (1.29–1.94)
	58–70	0.03	1.32 (1.07–1.63)
**Native of Corsica**	Native vs non-native	< 0.0000001	1.62 (1.41–1.86)

AOR: adjusted odds ratio; CI: confidence interval.

Age of reference: 38–47 years. The table includes only risk factors associated with p value < 0.05 in the multivariate analysis.

#### Age

**Figure 1:**
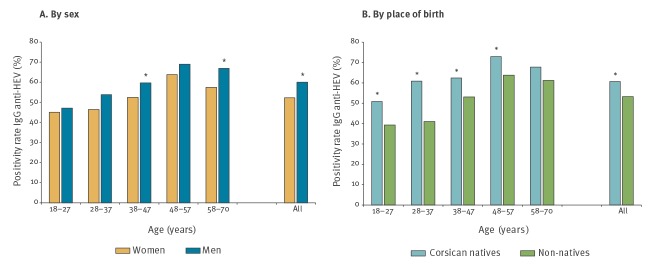
Distribution of anti-HEV IgG seroprevalence by age group, Corsica, 2017 (n = 2,705)

Among the 18–57-years-old study participants, the seroprevalence increased with age for both men and women (Figure 1A and Supplementary Figure S2A). The overall increase in seroprevalence was estimated at ca 0.62% and ca 0.72% per year in women and men, respectively (Supplementary Figure S2A). A rough estimate of the increase in seroprevalence in those too young to give blood was calculated between the ages of 1 year (as a proxy for the loss of maternal antibodies and for potential exposure to food-borne contamination) and 23 years (which corresponds to the median age of the 18–27-year age group): the mean yearly seroprevalence increase was 2.05% and 2.14% in women and men, respectively.

The asterisk indicates a statistically significant difference (p value < 0.05) between variables (sex in panel A and place of birth in panel B), for a given age group.

#### Corsican natives vs non-natives

The seroprevalence was higher in Corsican native individuals (60.6%) than in those born elsewhere (53.2%) in both univariate and multivariate analyses (Table 1 and 2). This difference was observed in all age groups (Figure 1B and Supplementary Figure S3). The increase in seroprevalence in the 18–57 years age group was estimated at ca 0.68% and ca 0.86% per year in native and non-native donors, respectively (Supplementary Figure S3). In those too young to give blood (1–23 years-old, see above), seroprevalence was estimated to increase annually by 2.31% and 1.78% in native and non-native individuals, respectively.

In the subpopulation of 18–57-year-old blood donors born on Corsica, the increase in seroprevalence was estimated at ca 0.60% and ca 0.76% per year in women and men, respectively (Supplementary Figure S2B). In those aged 1–23 years, the corresponding annual increase was 2.35% and 2.26% in women and men individuals, respectively.

#### Models of the annual force of infection

This increase in seroprevalence per year of age is expected to underestimate the annual probability of infection in susceptible individuals (i.e. the force of infection) because it does not account for the fact that only a proportion of an age group is susceptible for infection. For example, if the force of infection is 2% but only 50% of the age group is susceptible, the increase in the seroprevalence (1%) will substantially underestimate the force of infection (2%). The fact that the seroprevalence increases at a lower rate in adults than in children may therefore be partly explained by higher levels of susceptibility in children. We therefore used serocatalytic models to derive, from these data, estimates of the force of infection.

Restricting the analysis to native individuals, we found that the model that assumed a constant age-independent force of infection and no loss of immunity over time could not reproduce the large seroprevalence observed in the younger age group nor the plateau in the seroprevalence of older men (Supplementary Figure S4A and B). To integrate these features, we tested two alternative models (‘age-dependent’ force of infection vs ‘seroreversion’). Both models fit the data equally well. In the model where the force of infection was age-dependent (Supplementary Figure S4C and D), we found that the force of infection in children had to be 2.9 (95% CrI: 1.6–4.9) times higher than that in adults to explain the data (Table 3). Alternatively, a model assuming a long-term decay of immunity (average duration of immunity: 55 years; 95% CrI: 32–93) also provided a satisfying fit to the data as a loss of immunity in adults can explain the plateau in the age profile of seroprevalence (Supplementary Figure S4E and F). The estimated force of infection in that model was around 4% for men and women (Table 3).

**Table 3:** Parameter estimates for serocatalytic models of hepatitis E virus seroprevalence, Corsica, 2017

Models	Mean annual probability of infection in native susceptible men (95% CrI)	Mean annual probability of infection in native susceptible women (95% Crl)	Mean duration of immunity (range)
**Age-independent**	2.6% (2.3–2.8)	2.5% (2.3–2.8)	Indefinite
**Age-dependent <18 years**	3.9% (3.2–4.6)	3.7% (3.1–4.4)	Indefinite
**Age-dependent >18 years**	1.4% (0.89–1.9)	1.3% (0.8–1.9)	
**Seroreversion**	4.6% (3.6–6.2)	4.4% (3.4–5.8)	55 years (32–93)

Crl: credible interval.

Three models are considered. Age-independent: the force of infection is constant; age-dependent: the force of infection varies with age; seroreversion: the model includes decay of immunity.

### Anti-HEV IgG geographical distribution

Seroprevalence of anti-HEV IgG was analysed in the five Corsican administrative districts. Positive anti-HEV IgG rates were high, ranging from 53.22% in the Corte district to 58.26% in the Ajaccio district (Table 1 and Supplementary Figure S5)

### Anti-HEV IgG quantification

**Figure 2:**
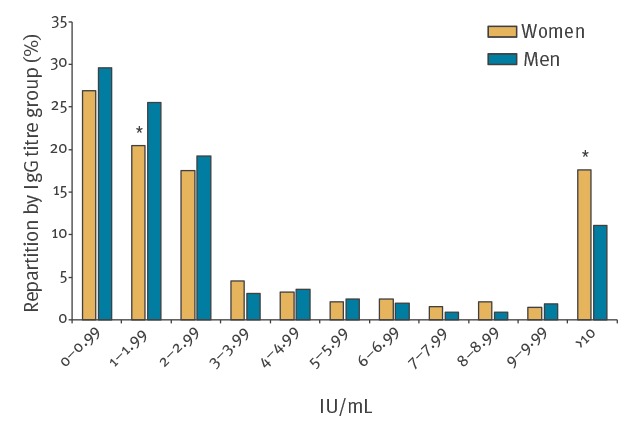
Distribution of anti-HEV IgG titre (IU/mL) estimated using the World Health Organization standard 95/584, by sex, Corsica, 2017 (n = 2,705)

All positive anti-HEV IgG samples (n = 1,518) were quantified. Titres ranged from 0.25 to 153 IU/mL, mean and median titres were estimated at 2.91 IU/mL and 0.46 IU/mL, respectively. Around 70% of positive blood samples had an anti-HEV IgG titre < 3 IU/mL and 85.90% had a titre < 10 IU/mL (Figure 2). Among the positive donors, women had a higher mean antibody titre (6.35 IU/mL) than men (4.17 IU/mL) (p < 0.05). Among women, 17.64% of positive samples had a titre > 10 IU/ml compared with 11.04% among men (p < 0.001). No difference was observed between titre and age groups (Supplementary Figure S6).

This asterisk indicates statistically significant difference, for a given titre, between sexes (p value < 0.05).

### Blood grouping

Among the 2,705 blood donors, the distribution of ABO blood groups was: O: 48.9% (n = 1,324), A: 37.7% (n = 1,019), B: 9.5% (n = 257) and AB: 3.9% (n = 105). Anti HEV IgG rates (Table 1) for ABO blood groups ranged from 52.92% (B) to 61.90% (AB). The prevalence values of Rhesus-positive (Rh D+ and Kell-positive (KEL +) phenotypes were 56.04% and 56.72%, respectively. No significant difference was observed when comparing the distribution of the anti-HEV IgG positive donations by blood groups or by combinations of them.

## Discussion

In 2011 and 2012, a nationwide survey (using the Wantai assay) identified geographical heterogeneity in the distribution of the anti-HEV IgG serological status among volunteer blood donors in mainland France, and Corsica Island was among the areas with the highest seroprevalence recorded (62%) [32]. The ficatellu, a local pork liver sausage, has been formally identified as a source of HEV food-borne contamination [45], but the actual impact of ficatellu consumption on HEV epidemiology is unknown and other potential sources of contamination remain to be explored. Here, we completed the abovementioned study by performing a specific analysis of a new and larger sample of Corsican blood donors.

Between September 2017 and January 2018 (a period without important touristic activity), we enrolled 2,705 blood donors residing on Corsica among whom 1,518 (56.1%) tested positive for anti-HEV IgG. This confirmed the high endemicity of HEV on Corsica. Other high prevalence areas have been identified in Europe using the same anti-HEV IgG assay, namely central Italy (Abruzzo region, 49%) [46] and south-western France (Midi-Pyrénées Region, 52.5%) [31].

The analysis of the raw data indicated that HEV seroprevalence is higher in men, a finding that was previously described in univariate but not multivariate analysis [32], and also higher in individuals born on Corsica. It was previously proposed that such differences may be explained by sociological factors (specific occupation, hunting, etc.), but there is to date no evidence-based explanation that takes into account the general high prevalence in both men and women and also the difference between the sexes. Association between human leukocyte antigen (HLA) or blood group antigens and infectious diseases has been documented in the literature [47-50] but has, to our best knowledge, never been investigated for HEV infection. Here, we did not identify such a link between ABO, Rhesus and Kell blood groups and the presence of anti-HEV antibodies.

Antibody titres are mostly low, with 77.2%, 81.6% and 85.9% of donors having anti-HEV IgG titres below 5, 7 and 10 IU/mL, respectively. The seroprevalence increases with age, except in those older than ca 60 years. In our models, the latter can be explained either by a force of infection that varies with age (but this is not supported to date by epidemiological or sociological information) or by a loss of specific antibodies over time. Previous studies have shown that antibody levels decrease over time and in some subjects, anti-HEV IgG can disappear after a follow-up ranging between 1 and 22 years [51]. A recent study among blood donors from central Italy using the Wantai assay has reported an HEV seroconversion rate of 2.1 per 100 person-years [52], in the same order of magnitude (1.3–4.6/100 person-years) as those estimated with our models.

Notably, the seroprevalence in the youngest age group of blood donors (18–27 years-old) was more than 10% higher in those born on Corsica (ca 51%) than in those born outside (ca 39%). This is reflected in a force of infection of 4.3% (95% CrI: 3.5–5.6) per year in natives vs 2.8% (95% CrI: 2.5–3.2) per year in non-natives in the ‘seroreversion model’. However, in the absence of detailed epidemiological data for individuals younger than 18 years, the exact shape of the seroconversion curve in children and teenagers remains to be established. Clearly, identifying determinants of exposure in the Corsican population younger than 18 years is essential to understand the epidemiology of HEV locally. A comparison of the HEV seroprevalence measured here (56.1%) with archived sera from blood donors collected in the year 2000 in both departments of Corsica (53.3%; n = 90) (data not shown) suggests that exposure to HEV has been stable in the Corsican population for at least two decades. In addition, we did not identify a significant variation in prevalence by administrative district of residence. Overall, the epidemiological information seems to converge towards a potential common, ubiquitous exposure to HEV infection for individuals living on Corsica.

The animal reservoir (mainly pigs and boars) is consensually regarded as a major source of HEV infection in humans [53,54]. However, the relative importance of direct (i.e. related to pig and boar meat consumption) or indirect routes of contamination remains unclear. Indirect contamination may be related to hand- or fomite-borne virus transmission, but also to contaminated drinking water. Previous information from the French nationwide study identified drinking bottled water as a protective factor against infection [32], and in the rural Auvergne region (central mainland France), water from the public network was identified as the common source of infection for a cluster of seven human cases, with HEV RNA detected in a private well that accidentally contaminated the public water network [55]. Moreover, a recent study in Sweden detected HEV genotype 3 strains in tap water and raw water before treatment [56]. Therefore, as more research is implemented to identify ubiquitous sources of HEV exposure on Corsica, the potential role of drinking water in the spread of HEV infection should be investigated.

## Conclusion

Our study confirms that Corsica is a high endemic area for HEV infection, with homogeneous exposure in the different geographical districts. Seroprevalence increases with age until 60 years and is higher in men than in women. Our study identified three priority fields for further investigations on Corsica. Firstly, the epidemiology in the younger age group (under 18 years) is essentially unknown in the absence of biological data and should be explored further. Secondly, common sources of contamination, in particular drinking water, deserve further studies because HEV can be found in faeces and wastewater [57] and Corsica is a region where infection of pigs and boars is frequent [58,59].The relationship between the animal reservoir, wastewater and the potential contamination of the public water network may be worth exploring. Thirdly, the high proportion of donors with low anti-HEV antibody titre raises questions about the protection afforded by IgG antibodies and about susceptibility to secondary HEV infection.

## Supplementary Data

1250000 Supplement
