# Supplementary material for: Seroprevalence of hepatitis E virus among blood donors on Corsica, France, 2017
Source: Euro Surveill. 2020 Feb 6;25(5):1900336. doi: 10.2807/1560-7917.ES.2020.25.5.1900336 (PMC7014670; doi:10.2807/1560-7917.ES.2020.25.5.1900336)
Supplement: Supplement [file 19-00336_CAPAI_Supplement.pdf]

Seroprevalence of Hepatitis E virus among blood donors in Corsica, France, 2017.

<u>Supplemental material</u>

Disclaimer: This supplementary material is hosted by *Eurosurveillance* as supporting information alongside the article *Seroprevalence of Hepatitis E virus among blood donors in Corsica, France, 2017*, on behalf of the authors, who remain responsible for the accuracy and appropriateness of the content. The same standards for ethics, copyright, attributions and permissions as for the article apply. Supplements are not edited by *Eurosurveillance* and the journal is not responsible for the maintenance of any links or email addresses provided therein.

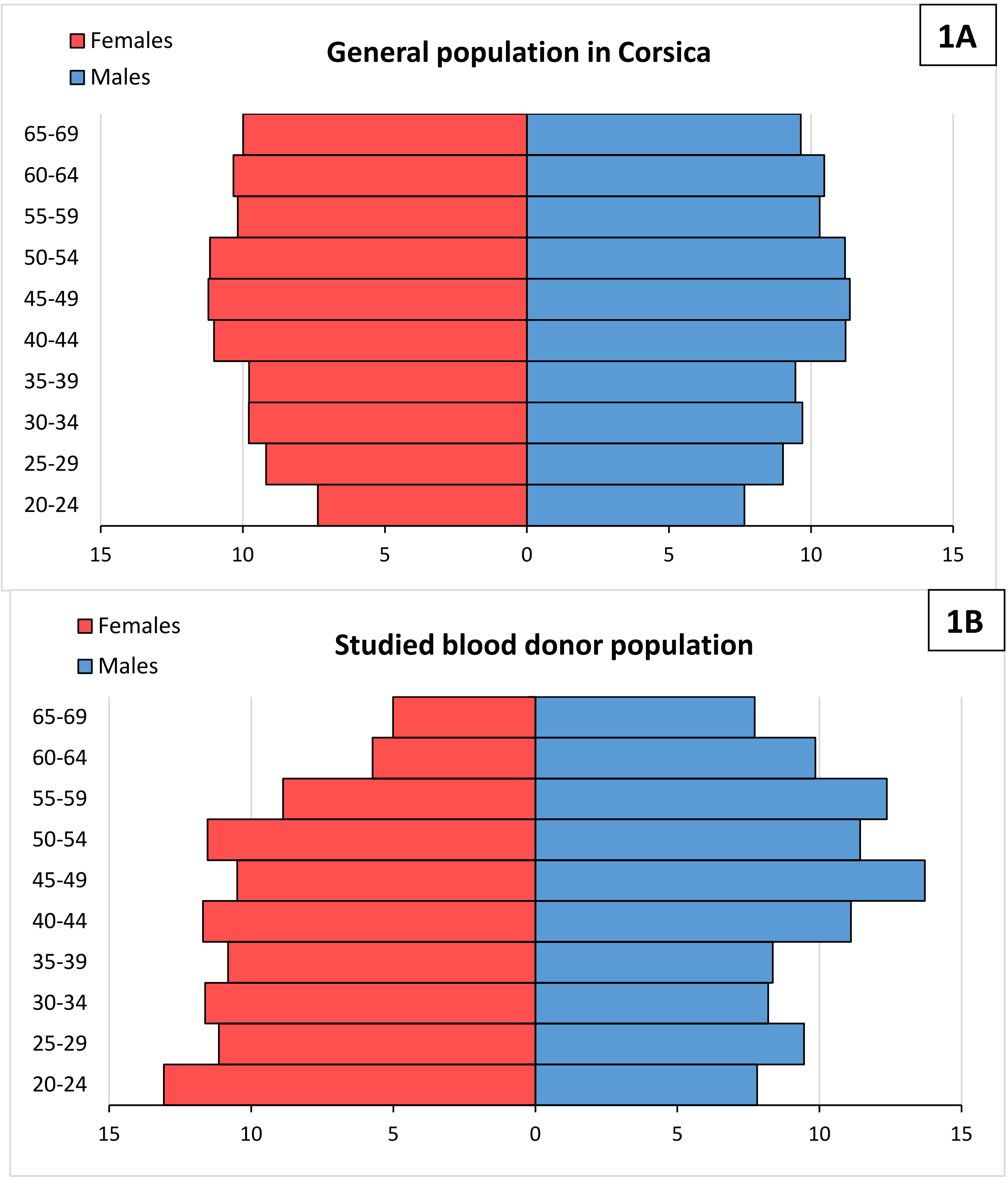

Supplemental Figure S1: demographic characteristics of general population in Corsica (1A) and the studied blood donor population (1B). Data in general population (2018) were obtained from the National Institute of Statistics and Economic Studies (INSEE), (https://www.insee.fr/fr/statistiques/3696315).

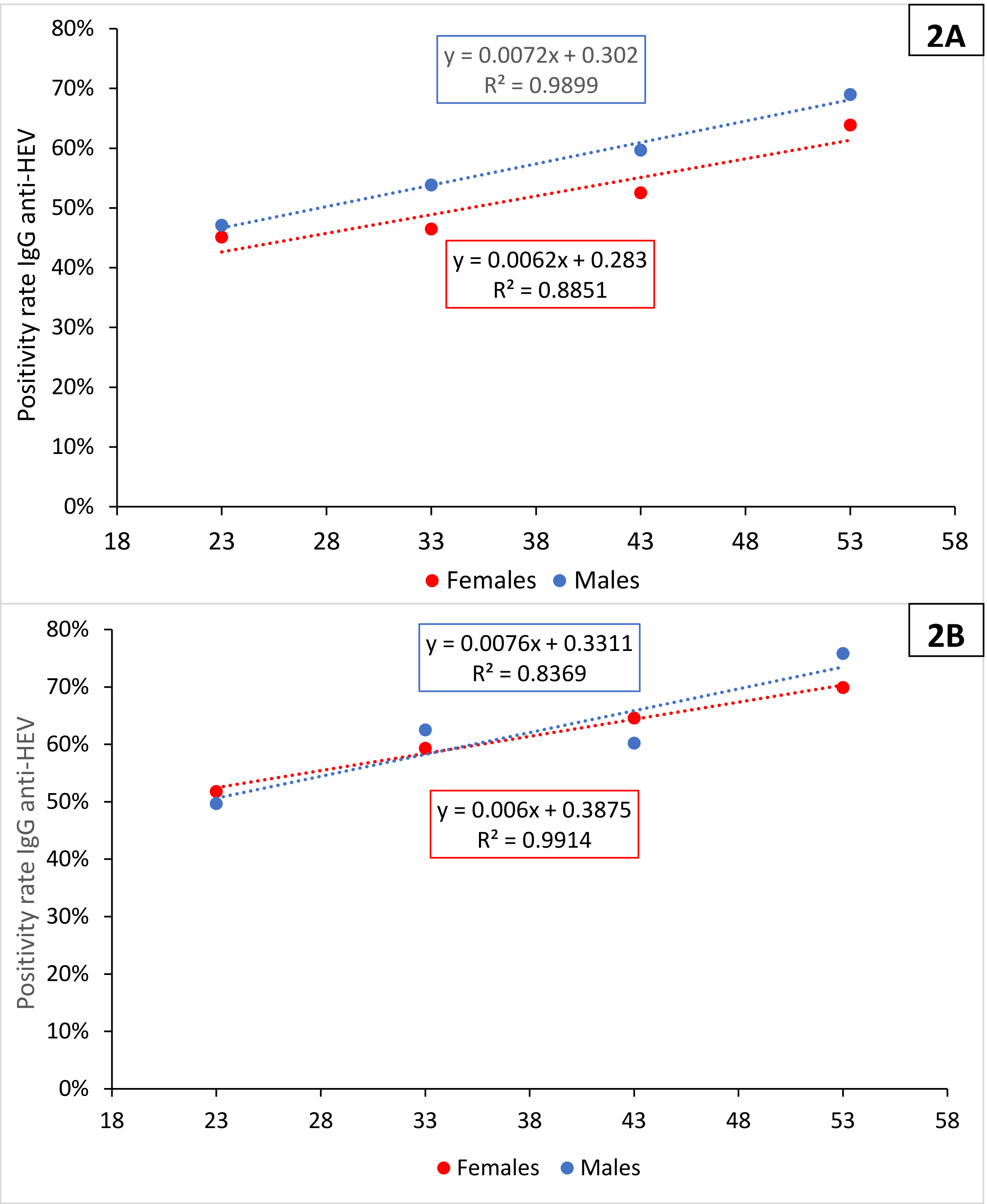

Supplemental Figure S2: HEV seroprevalence in age groups. Distribution by sex: red = females, blue = males. **Figure S2A:** all blood donors tested. **Figure S2B:** Natives of Corsica. In these graphs, in x axis, median age for each age group was used.

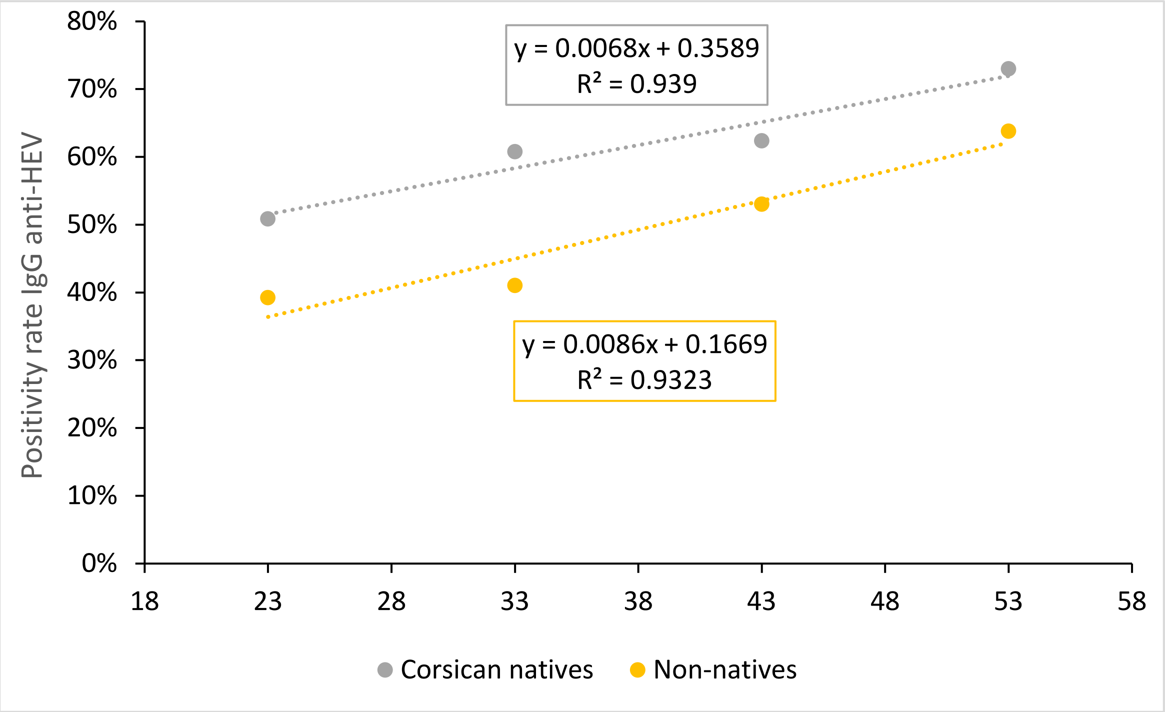

Supplemental Figure S3: HEV seroprevalence among native (grey) and non-native (yellow) of Corsica. In these graphs, in x axis, median age for each age group was used.

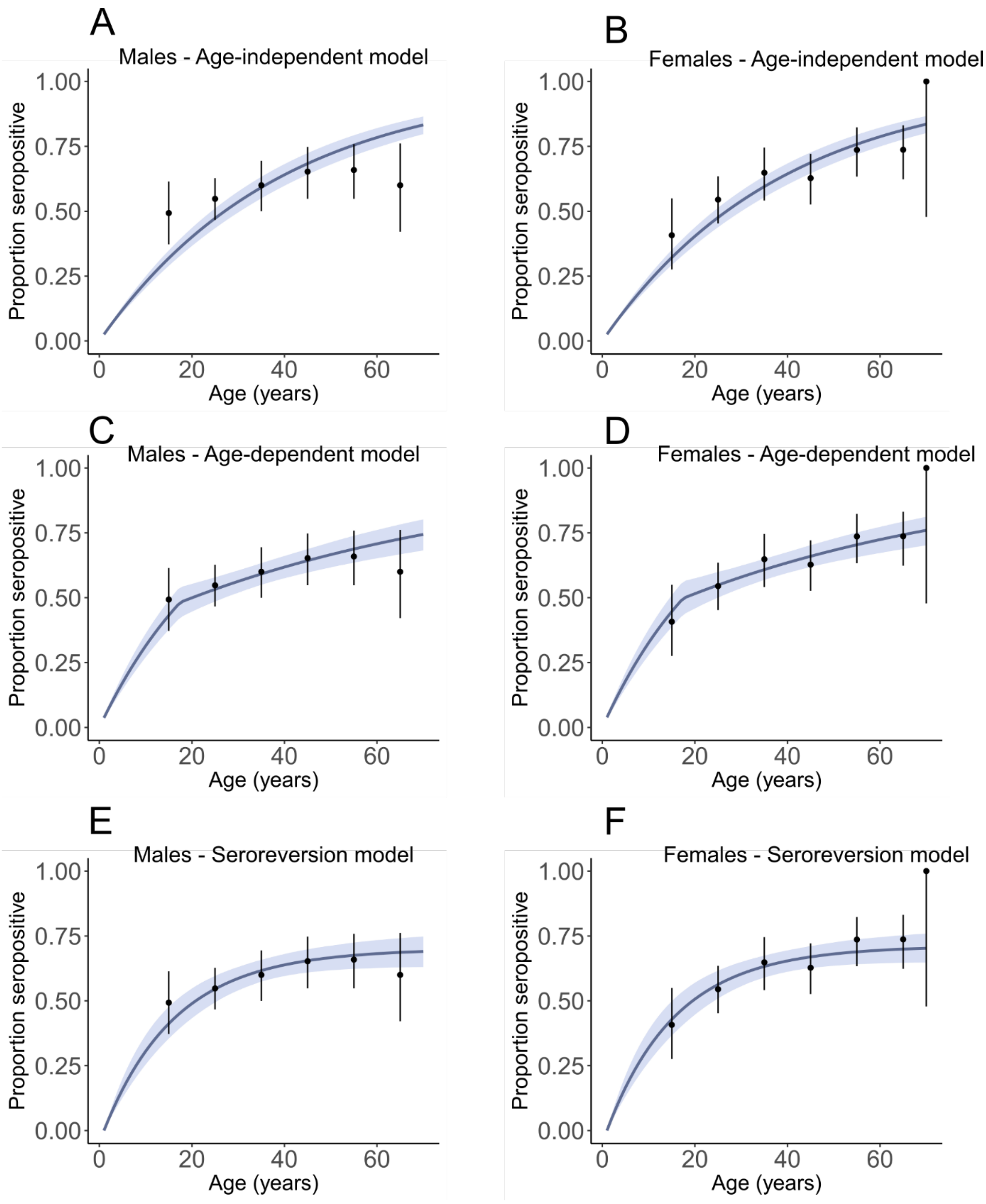

Supplemental Figure S4: Adequacy of serocatalytic models to age seroprevalence for native males and native females. We fit models where the force of infection is age-invariant ("age independent model", panels A & B) and age-dependent ("age-dependent model", panels C & D) as well as a reversible catalytic model ("seroreversion model", panels E & F). Black points and bars represent the mean and 95% binomial confidence intervals from the data, respectively. The solid blue line is the mean age seroprevalence obtained from the model fit and the envelope is the 95% credible interval.

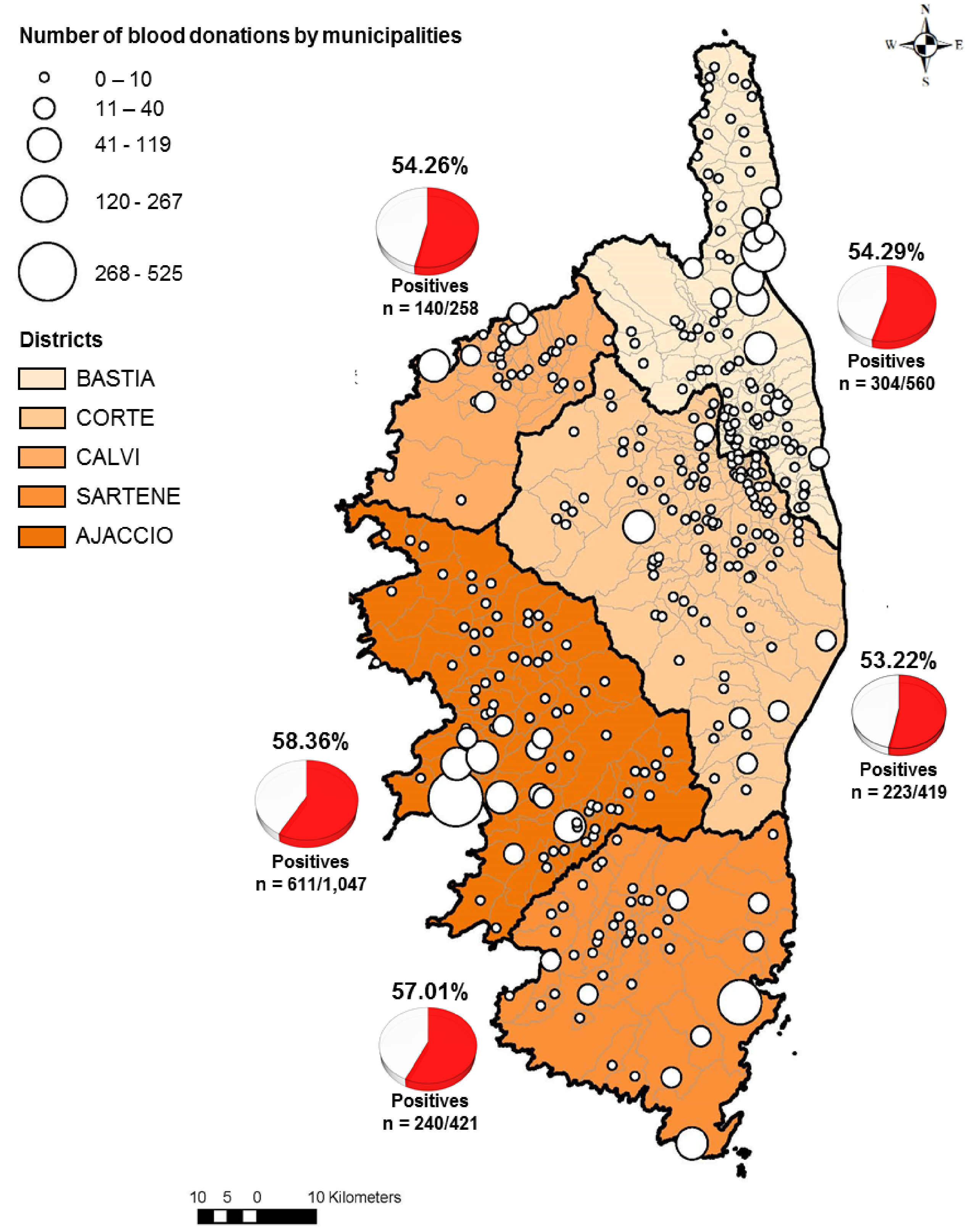

Supplemental Figure S5: Geographical distribution and number of blood donations (white circles) collected during the study according to the place of residence (municipality) and anti-HEV IgG seroprevalence in the 5 districts of Corsica (pie charts). The map was performed with the ARCGIS software (https://www.esri.com/arcgis/).

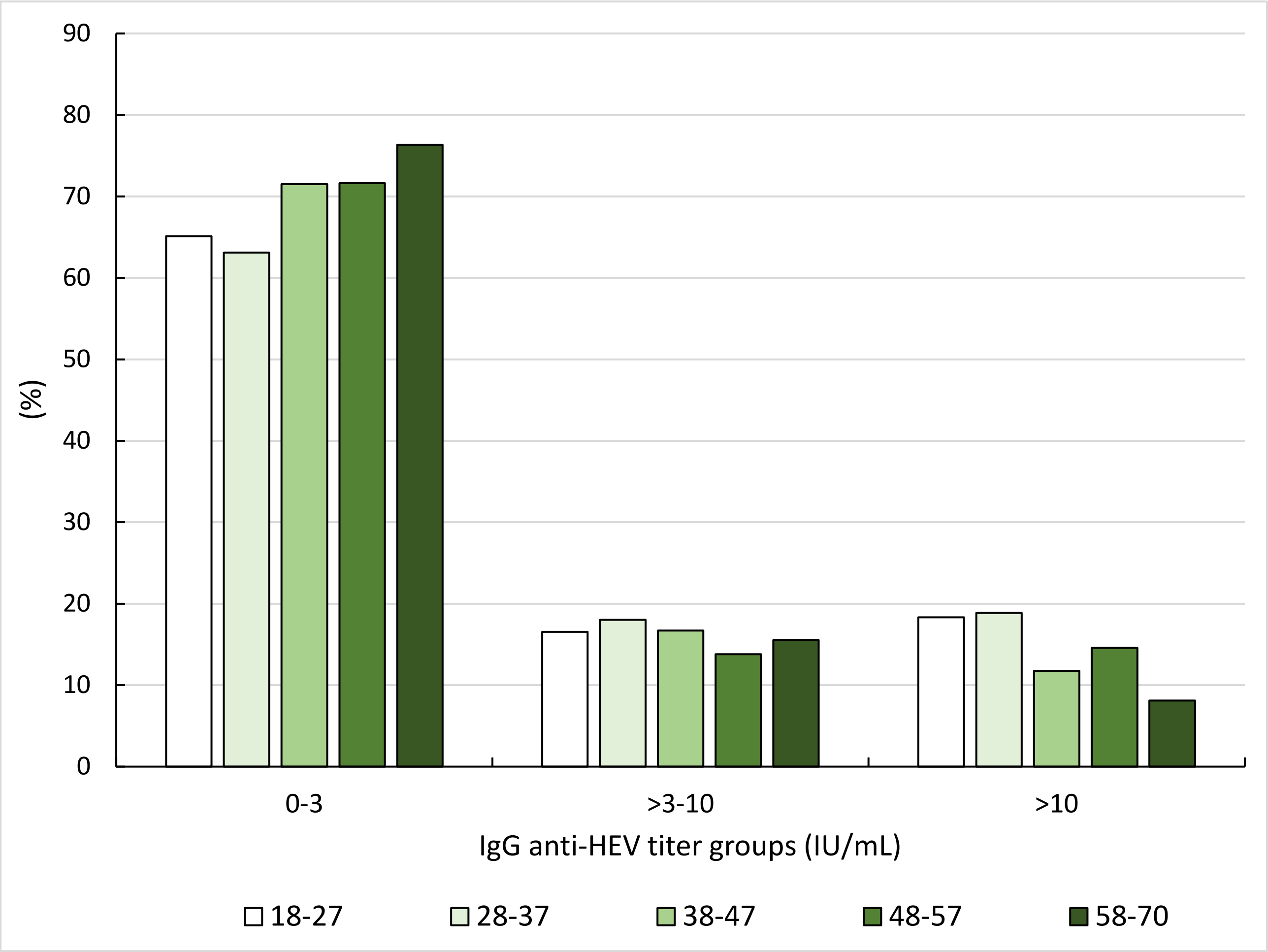

Supplemental Figure S6: Distribution of anti-HEV IgG titre (IU/mL) (estimated using the WHO standard 95/584) according to age groups.

Supplemental Table S1: Population by district in Corsica, adjusted numbers of donors by district and adjusted seroprevalence.

	Corsican population		Blood donor population of the study					
**District**	**n**	**Distribution by district**	**n**	**Distribution by district**	**Anti-HEV IgG Positive**	**Seroprevalence**	**Adjusted n**	**Anti-HEV IgG Positive after adjustment**
**Ajaccio**	113,618	34.15%	1,047	38.71%	611	58.36	924	539
**Bastia**	88,413	26.57%	560	20.70%	304	54.29	719	390
**Calvi**	29,842	8.97%	258	9.54%	140	54.26	243	132
**Corte**	59,183	17.79%	419	15.49%	223	53.22	481	256
**Sartene**	41,667	12.52%	421	15.56%	240	57.01	339	193
**All**	332,723	100.00%	2,705	100.00%	1,518	56.12	2,705	1510 (55.82%)
